# Picosecond mode switching and Higgs amplitude mode in superconductor-metal hybrid terahertz metasurface

**DOI:** 10.1515/nanoph-2022-0315

**Published:** 2022-08-09

**Authors:** Siyu Duan, Yushun Jiang, Jingbo Wu, Lu Ji, Ming He, Hongsong Qiu, Kebin Fan, Caihong Zhang, Guanghao Zhu, Xiaoqing Jia, Huabing Wang, Biaobing Jin, Jian Chen, Peiheng Wu

**Affiliations:** School of Electronic Science and Engineering, Research Institute of Superconductor Electronics (RISE), Nanjing University, Nanjing 210023, China; Purple Mountain Laboratories, Nanjing 211100, China; College of Electronic Information and Optical Engineering, Nankai University, Tianjin 300350, China; Key Laboratory of Photoelectronic Thin Film Devices and Technology of Tianjin, Tianjin 300350, China

**Keywords:** Higgs amplitude mode, metasurface, superconducting film, terahertz, ultrafast modulation

## Abstract

The ultrafast modulation of terahertz (THz) waves is essential for numerous applications, such as high-rate wireless communication, nonreciprocal transmission, and linear frequency conversion. However, high-speed THz devices are rare due to the lack of materials that rapidly respond to external stimuli. Here, we demonstrate a dynamic THz metasurface by introducing an ultrathin superconducting microbridge into metallic resonators to form a superconductor-metal hybrid structure. Exploiting the susceptibility of superconducting films to external optical and THz pumps, we realized resonance mode switching within a few picoseconds. The maximum on/off ratio achieved is 11 dB. The observed periodic oscillation of transmission spectra both in the time and frequency domain under intense THz pump pulse excitation reveals the excitation of Higgs amplitude mode, which is used to realize picosecond scale THz modulation. This study opens the door to ultrafast manipulation of THz waves using collective modes of condensates, and highlights an avenue for developing agile THz modulation devices.

## 1 Introduction

In the past decade, metasurfaces demonstrated versatile capabilities in manipulating electromagnetic waves [1–3] and found a variety of potential applications in holography [4–7], displays [8–10], and other fields [11–13]. Despite the impressive progress on static metasurfaces, metasurfaces capable of dynamically modulating electromagnetic waves are still in high demand for novel physical phenomena and applications [14, 15]. At terahertz (THz) frequencies, metasurfaces based on various tunable materials have been proposed for the dynamic manipulation of THz waves [16–19]. However, the devices with ultrafast THz response remain scarce, restricting their widespread application. In THz communication [20–23], high-speed modulators are essential for enhancing the data capacity to meet the booming requirement of next-generation wireless communication. For THz time-variant metasurfaces, the switching time must be comparable to the oscillation period of the THz wave, i.e., picosecond scale, to achieve nonreciprocal transmission [24, 25] and linear frequency conversion [26–28].

A feasible approach for the dynamic THz metasurface is embedding the tunable microstructures into metallic resonators to control the coupling between resonators and external stimuli. Thus, the spectral response can be rapidly switched [29, 30]. By periodically pinching off the conductive channels in semiconductor structures via field effects, the speed of semiconductor-based THz modulators can reach as high as the gigahertz regime [31–34]. However, the unavoidable parasitic effects and pronounced insertion loss of semiconductor microstructures make it challenging to further improve the THz modulation speed.

Superconducting film has been a fascinating choice of materials for active metasurface owing to its remarkable Ohmic loss and fast response to external stimuli near the phase transition temperature [35–39]. The high-quality factor and frequency tuning feature of the superconductor metasurface have been utilized to enhance the interaction between THz waves and matter [40–44]. Besides that, the energy gap of superconductors is in the order of meV, which is in the same order as the THz photon energy. When the superconducting film is illuminated with intense THz pulses, the resonant excitation can result in many interesting physical phenomena. Recently, the third-order nonlinear effect of the superconducting film under intense THz pulse was observed [45–47], and attributed to the resonant excitation between Higgs amplitude mode in the superconductor and the incident THz field [48, 49]. The Higgs mode, which refers to the collective amplitude fluctuation of the order parameter, reflects the macroscopic quantum nature of the superconductor [50, 51]. However, the Higgs mode has no coupling with the electromagnetic field in the linear response range, which raises difficulties in experimental observation and further application [52].

In previous studies on superconductor metasurface, resonators made from pure superconducting film were consistently employed [35, 53], [54], [55]. The pronounced resistance in the normal state and heat capacitance limits their sensitivity and response speed [35]. Using the tiny superconducting elements to control the coupling between the metallic resonators offers a reasonable alternative for developing high-speed THz modulation devices with a high on/off ratio. Furthermore, if the Higgs amplitude mode is excited in superconducting microstructures by a strong THz pump, the periodical coupling between metallic resonators will change with the oscillation of the order parameter, i.e., the superconductivity. This results in the ultrafast modulation of the THz spectral response.

Herein, we propose a superconductor-metal hybrid metasurface consisting of metallic resonators and ultrathin superconducting film microbridges. The superconducting microbridges control the coupling between metallic cavities through various stimuli. Employing pump-probe spectroscopy, we experimentally investigated the dynamic process of resonant mode switching in the hybrid metasurface under optical and THz pumps. The Higgs mode oscillation excited by the intense THz pump is studied in both temporal and spectral responses by altering the pump strength. Tuning the spectral response of coupled cavities through the superconductivity change in microbridges may offer an effective route for achieving high-speed THz modulation.

## 2 Scheme of hybrid metasurface

**Figure 1:**
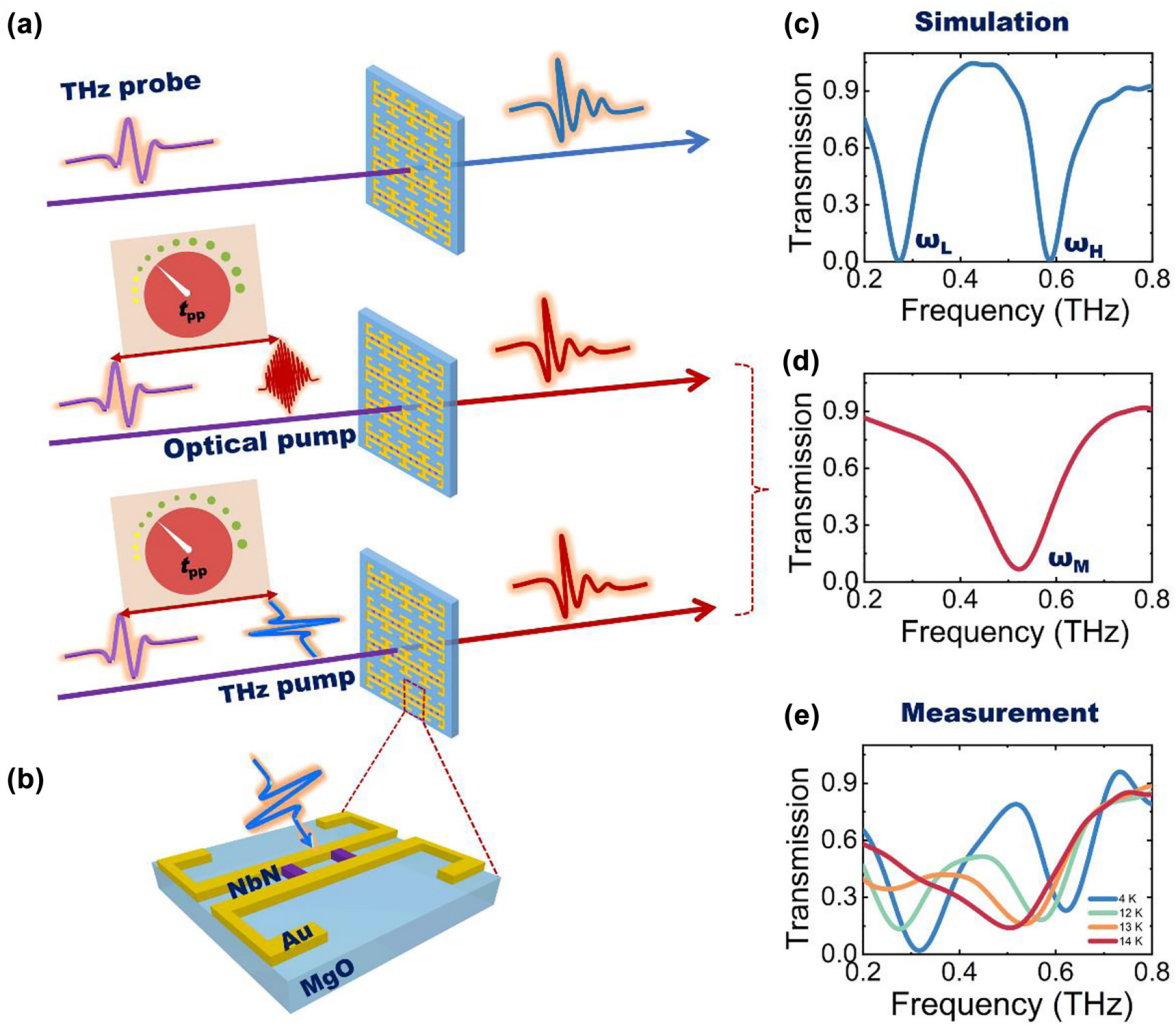
Schematic of THz transmission through hybrid metasurface under different pumps. (a) Schematic of THz probe pulse transmission through the superconductor-metal hybrid metasurface w/o pump or w/optical/THz pumps. Under optical and THz pumps, the pump-probe delay time (*t*_pp_) can be regarded as an adjustable knob. The corresponding simulated THz transmission spectra are shown in the right part. (b) Close-up of a unit cell of hybrid metasurface under the THz pump. (c) and (d) Simulated THz transmission spectra without and with pump. (e) Measured THz transmission spectra at different temperatures.

The transmission of the THz probe pulse through the proposed superconductor-metal hybrid metasurface is schematically shown in Figure 1. We studied the ultrafast switching process of THz transmission spectra using the pump-probe spectroscopy system. As shown in Figure 1(a), THz spectral responses were studied under three configurations: without pump, with optical pump, and with THz pump. As illustrated in Figure 1(b), the unit cell structure consists of an array of double niobium nitride (NbN) microbridges connected with the mirror-symmetrical metallic split-ring resonators (SRR) (see Supplementary Note 1 for details). The electric field direction of the incident THz wave is perpendicular to the gap. Using the dramatic change of NbN conductivity during the phase transition, the conductive coupling between two resonators can be altered. Based on the simulation results, the conductive coupling between two resonators is critical for mode switching [56] (see Supplementary Note 3 for details). After design optimization, we chose 12-nm thick NbN film to fabricate microbridges.

The simulation results w/o and w/pump are shown in Figure 1(c) and (d). The transmission coefficient was obtained based on *T*(*ω*) = *E*_S_(*ω*)^2^/*E*_R_(*ω*)^2^, where *E*_S_(*ω*) and *E*_R_(*ω*) are Fourier transformed spectra of the THz pulses transmitting through the sample and bare substrate, respectively. When the pump pulse is absent, there are two resonant modes in the simulated transmission spectra. Two resonance dips at 0.28 THz (marked as *ω*_L_) and 0.59 THz (marked as *ω*_H_) correspond to the charge transfer plasmonic (CTP) and the screened bonding dimer plasmonic (SBDP) modes in the plasmonic dimers [29, 57, 58]. When optical pulse or intense THz pump pulses are applied, the phase transition from the superconducting to the normal state could be induced in microbridges. Only one resonance valley appears at 0.52 THz (marked as *ω*_M_). The resonance mode is similar to the bonding dimer plasmonic (BDP) mode in plasmonics. The simulated electric field distribution of the metasurface at 0.55 THz normalized by the bare substrate is plotted in Figure S1c. There is no significant field enhancement effect around NbN microbridges, demonstrating that the role of a superconducting microbridge is to control the coupling between metallic cavity resonators.

The measured THz transmission spectra at different temperatures are shown in Figure 1(e). The experimental results are consistent with the simulation results. At 4 K, there are two resonance valleys at 0.32 and 0.62 THz, respectively. As the temperature increases, the two resonance frequencies gradually redshift. When the temperature rises to 14 K, mode switching occurs, and the BDP resonance mode appears at 0.51 THz. Before mode switching, there is remarkable frequency tuning for the CTP and SBDP resonance modes. The frequency tuning property can be attributed to the temperature-dependent kinetic inductance of the NbN microbridge in the superconducting state [41] (see Supplementary Note 4 for details).

## 3 Ultrafast mode switching in hybrid metasurface

**Figure 2:**
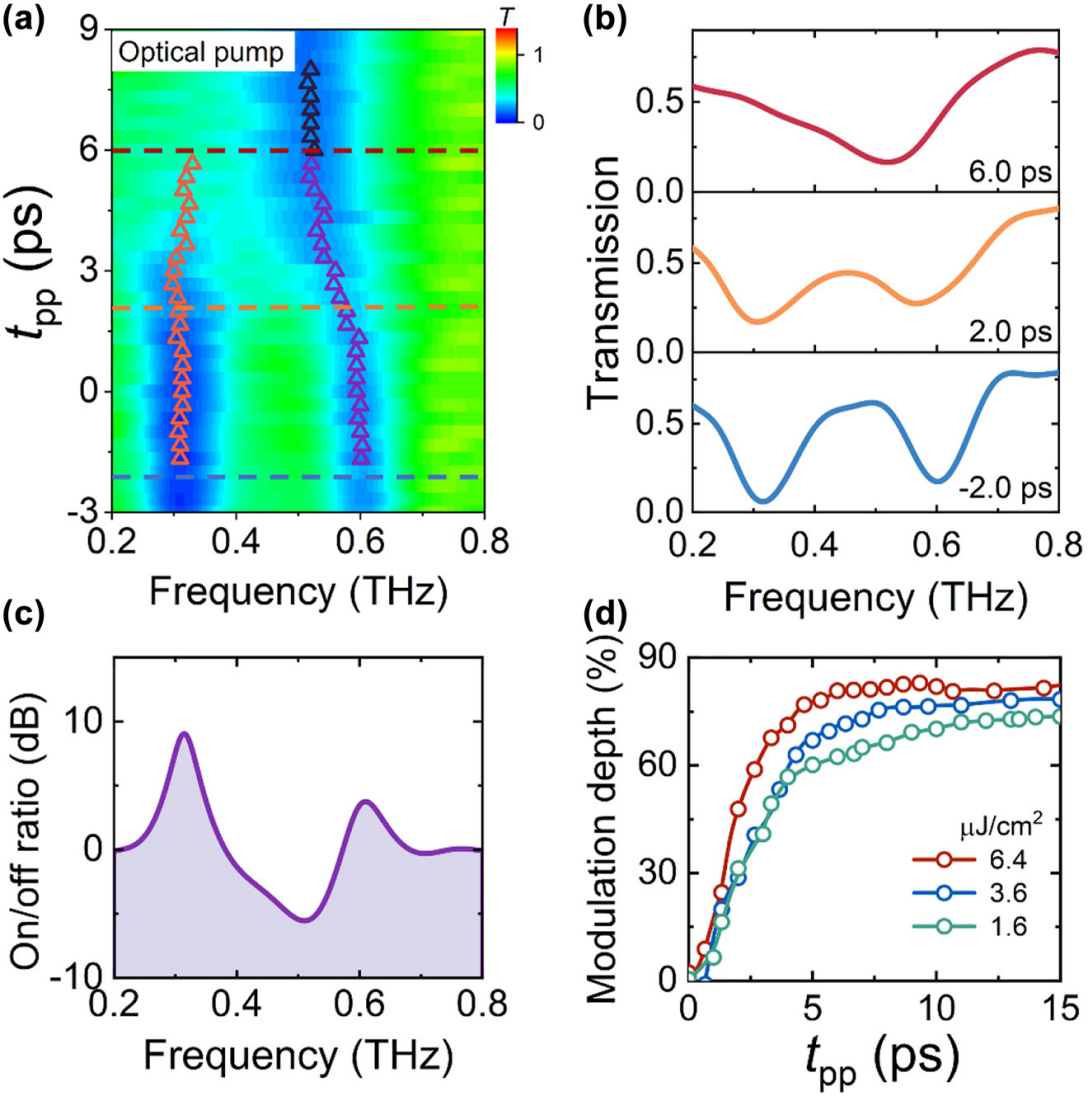
Ultrafast mode switching triggered by optical pump. (a) Mapping of measured transmitted spectra as a function of *t*_pp_ at optical pump fluence of 6.4 μJ/cm^2^. The resonance frequency is marked with triangles. (b) Measured THz transmission spectra at *t*_pp_ = −2.0, 2.0, and 6.0 ps, corresponding to the dashed lines marked in (a). (c) Measured on/off ratios as a function of frequency at 4 K at optical pump fluence of 6.4 μJ/cm^2^. (d) Measured modulation depth at 0.32 THz as a function of *t*_pp_ under different pump intensities.

The experimental diagram for optical pump-THz probe measurement is shown in the center of Figure 1(a). The center wavelength of the optical pulse is 800 nm, and the pulse width is 100 fs. When the optical pump fluence is 6.4 μJ/cm^2^, the mapping of the transmission spectra as a function of the pump-probe delay time (*t*_pp_) at 4 K is shown in Figure 2a. The resonance frequencies at different *t*_pp_ are marked with triangles. The time that the electric field strength starts to change at *t*_gate_ = 6.7 ps is defined as *t*_pp_ = 0 ps, rather than the moment when the main peaks of the pump and probe pulses are aligned (see Supplementary Note 5 for details). The spectra at *t*_pp_ of −2.0, 2.0, and 6.0 ps are shown in Figure 2(b), denoted by dotted lines in Figure 2(a). When the optical pump is applied, the superconductivity of microbridges is gradually suppressed by optical pump pulses, resulting in the gradual change of the transmission spectra when *t*_pp_ ≥ 0 ps. At *t*_pp_ ∼2.0 ps, the change of the spectra is the most significant. It is probably corresponding to the moments when the main peaks of the pump and probe pulse are aligned. The two resonance modes finally merge into one resonance mode at *t*_pp_ = 6.0 ps.

The photon energy of the optical pump is 1.55 eV, significantly higher than the NbN gap energy of 5.2 meV. The absorption of photons results in the breaking of Cooper pairs and the generation of quasiparticles, thereby suppressing superconductivity. The thermalization process involving quasiparticles, phonons, and Cooper pairs typically takes a few picoseconds to reach a quasi-stable state based on recent studies [59, 60]. In our experiment, the dynamic mode switching process in the measured time evolution of THz transmission spectra (Figure 2(a)) reflects the suppression of superconductivity in NbN microbridges.

The mode switching yields a large on/off ratio, which is a crucial indicator for the THz modulator. The on/off ratio (*к*) is defined as *к* = 10 ∙ log(*T*_on_/*T*_off_), where *T*_on_ and *T*_off_ are the transmission coefficients before and after mode switching, respectively. Using the measured transmission spectra at *t*_pp_ = −2.0 ps and 6.0 ps, we calculated the on/off ratio spectra as shown in Figure 2(c). The highest value is approximately 9 dB at 0.32 THz for the CTP resonance mode. The on/off ratio is as high as 6 dB at 0.51 THz for the BDP mode and 4 dB at 0.61 THz for the SBDP mode.

According to previous studies, the conductivity changes of superconducting films under optical pumping depend on the pump strength. The transmission spectra and mode switching time of the hybrid metasurface can also be tuned by the pump intensity, as shown in Figure S5. Because the on/off ratio is the largest at around 0.32 THz, the modulation depth (*η*) at different pump fluences is calculated as *η* = ((*T*(*t*_pp_) – *T*(*t*_pp_=0))/*T*(*t*_pp_)) ∙ 100%. As shown in Figure 2(d), *t*_pp_ can be regarded as a knob adjusting the resonance amplitude of the transmission spectra. When the pump energy density is 6.4 μJ/cm^2^, the modulation depth is as high as 90%, and the mode switching is completed in 6 ps. Though the mode switching is not realized at a pump energy density of 1.6 μJ/cm^2^ due to the relatively smaller conductivity change, the modulation depth still reaches 73%, which is attributed to the weakening of resonance strength.

In the following, we performed the THz pump-THz probe measurement. The diagram of the experimental setup is shown at the bottom of Figures 1(a) and S2. In the experiment, the polarization direction of the THz pump is perpendicular to that of the detection crystal. The maximum pump THz electric field strength (*E*_0_) is 25 kV/cm. The time-domain profile of THz pump pulse and corresponding Fourier transformed frequency spectra are shown in Figure S6. Within the experimental error range, there was no leakage of the THz pump pulse into the waveform of the probe electric field.

**Figure 3:**
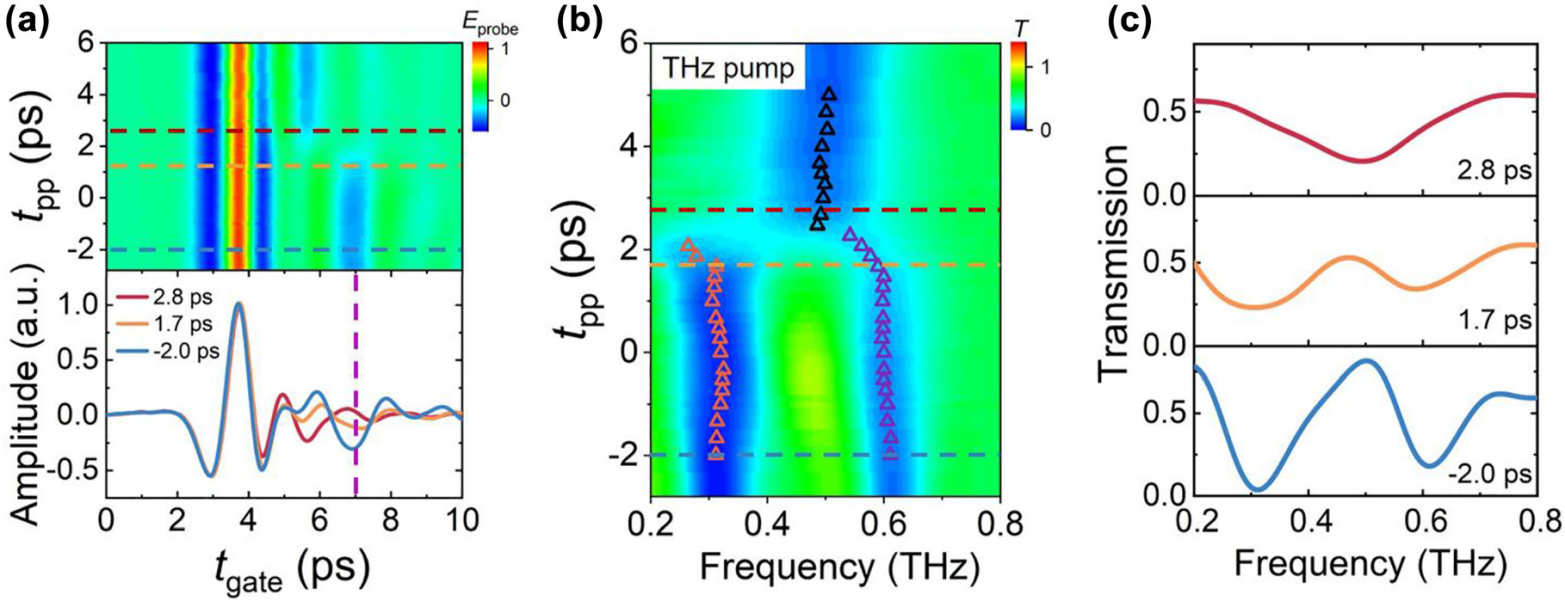
Ultrafast mode switching triggered by intense THz pump pulse. Two-dimensional plot of the transmitted THz pulse profile (a) and spectra (b) as a function of *t*_pp_ when the *E*_0_ is 25 kV/cm. The resonance frequencies are marked with triangles. (c) THz transmission spectra at *t*_pp_ = −2.0, 1.7, and 2.8 ps corresponding to dashed lines marked in (a) and (b).

The mappings of the transmitted THz pulse and spectra at 4 K as a function of *t*_pp_ are plotted in Figure 3(a) and (b). The time-domain signal is normalized by the peak of the main pulse. There was no apparent amplitude change and temporal shift for the main pulses around *t*_gate_ = 3.5 ps in the time domain. On contrary, the trailing signals around *t*_gate_ = 5.7 and 7.0 ps exhibit drastic changes. Unlike pure superconducting film, the THz pump only causes significant changes in the electromagnetic responses near the resonant modes of the hybrid metasurface, but not in the entire frequency band. The time of electric field intensity change at *t*_gate_ = 7.0 ps is defined as *t*_pp_ = 0 ps. As shown in Figure 3(b), there are apparent changes in the measured transmission spectra when the THz pump is applied. The resonance frequencies at different *t*_pp_ are marked with triangles. Similar to the optical pump, mode switching from two resonant modes to a single mode occurs, and the transition process takes ∼2.5 ps. This means that the THz pump can suppress the superconducting state of the NbN microbridges. The transmission spectra at the *t*_pp_ of −2.0, 1.7, and 2.8 ps are shown in Figure 3(c), denoted by the dotted lines in Figure 3(a) and (b). The maximum on/off ratio is likewise approximately 11 dB.

As shown in Figure S6, the frequency spectra of the THz pump pulse span 0.2–1.2 THz, and the peak field is concentrated around 0.5 THz (2 meV), which is lower than the NbN gap frequency of 1.2 THz (5.2 meV). To explore the physical mechanism of mode switching under THz pump pulse excitation, we calculated the ponderomotive energy (*U*_p_) using the following equation [61]

(1) $$U_{\text{p}}=\frac{{\text{e}}^{2}E^{2}}{4m_{0}\omega^{2}}$$

where *e* is the electron charge, *E* is the peak THz electric field, *m*_0_ is the electron mass, and *ω* is the angular frequency. When the frequency of THz pulse is 0.5 THz and *E* is 25 kV/cm, the calculated *U*_p_ is about 28 meV, which is sufficient to break Cooper pairs. In this case, the THz pump pulse with the intense electric field could break up Cooper pairs after *t*_pp_ = 0 ps.

### 4 Higgs amplitude mode excitation under THz pump

**Figure 4:**
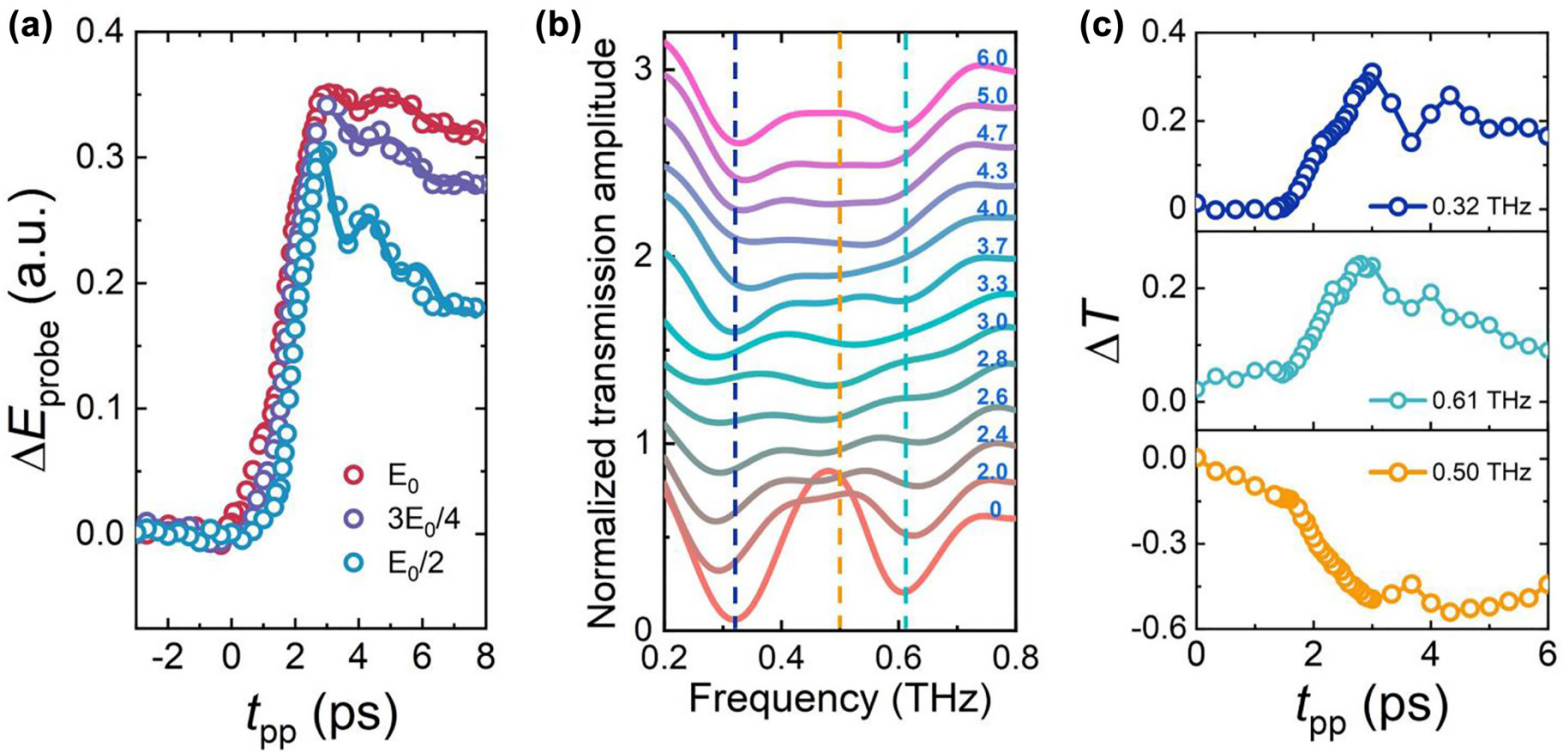
Mode switching dynamics and Higgs amplitude mode excitation under THz pump. (a) Measured temporal evolution of Δ*E*_probe_ (open circles) as a function of *t*_pp_ = under different pump THz field strength (*t*_gate_ = 7.0 ps), corresponding to the purple dashed lines marked in Figure 3(a). The fitting curves are represented by solid lines. (b) Measured normalized THz transmission spectra for different *t*_pp_ when the pump field strength is *E*_0_/2 (each curve is offset vertically by 0.2). (c) Measured transmission change relative to the transmission coefficient at *t*_pp_ = 0 s at 0.32, 0.61, and 0.5 THz as a function of *t*_pp_ when the pump field strength is *E*_0_/2.

According to previous measurements on superconducting films, the oscillation of the Higgs amplitude mode is observed in the measured temporal evolution of Δ*E*_probe_ under intense THz pump pulse excitation [45–49], where Δ*E*_probe_ refers to the change of electric field of the THz probe pulse (*E*_probe_). In our experiment, we also investigate the temporal evolution of Δ*E*_probe_ at *t*_gate_ = 7.0 ps for different THz pump strengths, as shown in Figure 4(a). We chose *t*_gate_ = 7.0 ps, because the amplitude change is the most drastic, purple dashed lines marked in Figure 3(a). The temporal evolution of Δ*E*_probe_ at *t*_gate_ = 5.7 ps is also measured (see Supplementary Note 7). After the incidence of the THz pulse pump, Δ*E*_probe_ increases sharply. The step-like rise signal indicates that the superconductivity is suppressed instantaneously. A stronger THz pump intensity leads to a higher rise in the signal. After the initial overshoot of Δ*E*_probe_, we could see an apparent drop at *t*_pp_ = 3.0 ps. The oscillation signals appear after the falling edge and last until *t*_pp_ = 6.0 ps. Based on the simulation results, the oscillations indicate that the property of superconducting microbridge undergoes evident oscillatory changes. In the previous experimental results of pure NbN/Nb_1−*x*_Ti_*x*_N film [46, 62, 63], the amplitude oscillation of the order parameter is manifested in the oscillation of the conductivity after the THz pump. By comparing it with the reported results, we infer that the oscillations may be attributed to the excitation of the Higgs amplitude mode in the NbN microbridge.

To further verify the presence of the Higgs amplitude mode, we analyze the fluctuations in the spectral responses of the hybrid metasurface and extract the frequency of oscillations at different pump intensities. The oscillating part of Δ*E*_probe_ is fitted by the following equation [48],

(2) $$\Delta E_{\text{probe}}\left(t_{\text{pp}}\right)=C_{1}+C_{2}t_{\text{pp}}+a\frac{\cos\left(2\pi \;f{\;t}_{\text{pp}}+\varphi \right)}{{\left(t_{\text{pp}}-t^{\prime }\right)}^{b}}$$

where *C*_1_, *C*_2_, *a, b*, *φ*, and *t*′ are parameters. The fitting curves (solid lines) are shown in Figure 4(a), and the extracted the oscillation frequency *f* at various pump intensities are listed in Table 1. The Higgs amplitude mode frequency tends to decrease with increasing pump strength, which is consistent with the results of previous pure film measurements. With the increase in the THz pump intensity, the proportion of Cooper pairs being broken up increases, leading to a decrease in the energy gap frequency after the transition process [48].

**Table 1:** Extracted Higgs mode frequencies under different THz pump intensity.

Pump intensity	*E*_0_	3*E*_0_/4	*E*_0_/2
Higgs mode frequency (THz)	0.347	0.426	0.62

In the following, we analyze the corresponding frequency spectra under different pump strengths. Because the fluctuations are more remarkable under the weaker pump field, we plot the measured transmission spectra from *t*_pp_ = 0 – 6.0 ps when the pump strength is 1/2*E*_0_, as shown in Figure 4(b). Taking the transmission of *t*_pp_ is 0 ps as a reference, the change in the transmission (Δ*T*) at 0.32, 0.61, and 0.5 THz as a function of *t*_pp_ are plotted in Figure 4(c). The Δ*T* of the three frequencies varies non-monotonously with *t*_pp_, which exhibits a similar oscillatory behavior to Δ*E*_probe_ in the region from 3.0 to 6.0 ps. At frequencies far from the three resonance modes, there is almost no oscillation for Δ*T*. The frequency spectra from *t*_pp_ = 0 – 6.0 ps and Δ*T* at three resonance frequencies as a function of *t*_pp_ when the pump strength is 3*E*_0_/4, or *E*_0_ are plotted in Figure S8. Under different pump strengths, the Δ*T* also exhibits similar oscillation behaviors. A stronger oscillation of Δ*E*_probe_ leads to a more pronounced modulation of the transmission amplitude.

## 5 Discussion

We observed the excitation of the Higgs amplitude mode in the hybrid metasurface under the THz pump. The conductivity fluctuation in superconducting microbridges results in the oscillation of the transmission spectra with *t*_pp_, particularly for the transmission amplitudes around resonance frequencies. The spectral response of the metasurface functions as an “indicator” of the Higgs mode. We can monitor the changes in the Higgs amplitude mode based on the resonant strength and mode switching of the cavity. More importantly, we can apply the Higgs amplitude mode excitation to control the coupling between metallic cavities. Because the Higgs amplitude mode has no coupling interaction with the cavity modes, and its frequency is in the THz range, the hybrid metasurface provides an excellent platform to develop an ultrafast THz modulator.

Furthermore, the proposed hybrid meta-device still has room for improvement in terms of the key indicators of the THz modulator. The working frequency of the modulator can be altered by changing the resonance frequencies of metallic cavities. The modulation speed is determined by the Higgs mode oscillation period. The Higgs mode frequency shows dependence on temperature, electric bias, and THz pump strength. Thus, the modulation frequency is tunable by a variety of means. In this study, the obtained modulation depth is not high due to the weak Higgs mode oscillation. By optimizing the cavity design and conditions for the excitation of Higgs amplitude mode, the modulation depth could be further improved.

In summary, we experimentally demonstrated an ultrafast and frequency-agile NbN-Au hybrid THz metasurface. The dynamic manipulation of the spectral response was realized by triggering the resonant mode switching with thermal, optical, and THz stimuli. Moreover, the THz pump-THz probe experiment shows picosecond scale oscillations in both the time and frequency spectra, which can be attributed to the fluctuation of the coupling coefficient induced by the Higgs amplitude mode. The application of superconducting order parameter oscillation for ultrafast THz modulation offers an entirely novel scheme for the development of high-performance THz devices, which may extend to other phase change materials.

## 6 Methods

### 6.1 Device fabrication

First, the 12 nm thick NbN film was deposited on the 500 μm thick MgO substrate using radio frequency magnetron sputtering. The measured superconducting transition temperature was 13.5 K. Then, the NbN bridge was patterned by ultraviolet photolithography and reactive ion etching processes (with a mixture of SF6 and CHF3). The complementary pattern of split-ring resonators was formed using photolithography. The 200 nm thick gold film was deposited using magnetron sputtering. After the lift-off process, the metallic resonators were formed.

### 6.2 Electromagnetic simulation

Using the Born limit impurity scattering model based on the BCS theory, the complex conductivity of the NbN film was calculated at different temperatures. The permittivity with dispersion characteristics was obtained at different temperatures. The time-domain solver of the electromagnetic simulation software was used to calculate the transmission spectra at different temperatures. The substrate used in the simulation was MgO substrate, and the permittivity was set to be 9.8.

### 6.3 THz spectroscopy measurement

The cryogenic THz time-domain spectroscopy system was used to characterize the transmission spectra of the metasurface. The metasurface was placed in a helium-free cryostat with optical windows. The THz transmission spectra represent the ratio of the Fourier transformed spectra of the time-domain signals through the sample and the reference of bare MgO substrate. The cryogenic THz/optical pump-THz probe system was used to measure the pump-probe spectra of the metasurface (see Supplementary Note 2 for experimental setup). By changing the optical path difference between the THz probe pulse and pump pulse, the *t*_pp_ between the two pulses can be controlled. By changing *t*_gate,_ the time-domain profiles of the transmitted THz probe pulse could be obtained.

## Supplementary Material

Supplementary Material Details
