# Supplementary material for: Picosecond mode switching and Higgs amplitude mode in superconductor-metal hybrid terahertz metasurface
Source: Nanophotonics. 2022 Aug 9;11(18):4253–61. doi: 10.1515/nanoph-2022-0315 (PMC11501531; doi:10.1515/nanoph-2022-0315)
Supplement: Supplementary file 1 — Supplementary Material Details [file j_nanoph-2022-0315_suppl.docx]

Supporting information

Picosecond mode switching and Higgs amplitude mode in superconductor-metal hybrid terahertz metasurface

Siyu Duan, Yushun Jiang, Jingbo Wu*, Lu Ji*, Ming He, Hongsong Qiu, Kebin Fan, Caihong Zhang, Guanghao Zhu, Xiaoqing Jia, Huabing Wang, Biaobing Jin*, Jian Chen, Peiheng Wu

***Corresponding authors:** **Jingbo Wu, Biaobing Jin,** Research Institute of Superconductor Electronics (RISE), School of Electronic Science and Engineering, Nanjing University, Nanjing 210023, China; Purple Mountain Laboratories, Nanjing 211100, China, Email: [jbwu@nju.edu.cn](mailto:jbwu@nju.edu.cn), [bbjin@nju.edu.cn](mailto:bbjin@nju.edu.cn). **Lu Ji,** College of Electronic Information and Optical Engineering, Nankai University, Tianjin 300350, China; Key Laboratory of Photoelectronic Thin Film Devices and Technology of Tianjin, Tianjin 300350, China, Email: luji@nankai.edu.cn

**Siyu Duan, Yushun Jiang, Hongsong Qiu, Kebin Fan, Caihong Zhang, Guanghao Zhu, Xiaoqing Jia, Huabing Wang, Jian Chen, Peiheng Wu,** Research Institute of Superconductor Electronics (RISE), School of Electronic Science and Engineering, Nanjing University, Nanjing 210023, China

**Kebin Fan, Caihong Zhang, Xiaoqing Jia, Huabing Wang, Jian Chen,** Purple Mountain Laboratories, Nanjing 211100, China
**Ming He,** College of Electronic Information and Optical Engineering, Nankai University, Tianjin 300350, China; Key Laboratory of Photoelectronic Thin Film Devices and Technology of Tianjin, Tianjin 300350, China

This document contains supplementary information on “Picosecond cavity-mode switching modulated by Higgs amplitude mode in superconductor-metal hybrid metasurface.” We provide details on the structure design, light path, experimental method, and additional analysis results.

Pages S1-S8, 8 figures.

**Supplementary Note 1 Diagram and simulated electric field distribution of the hybrid metasurface**


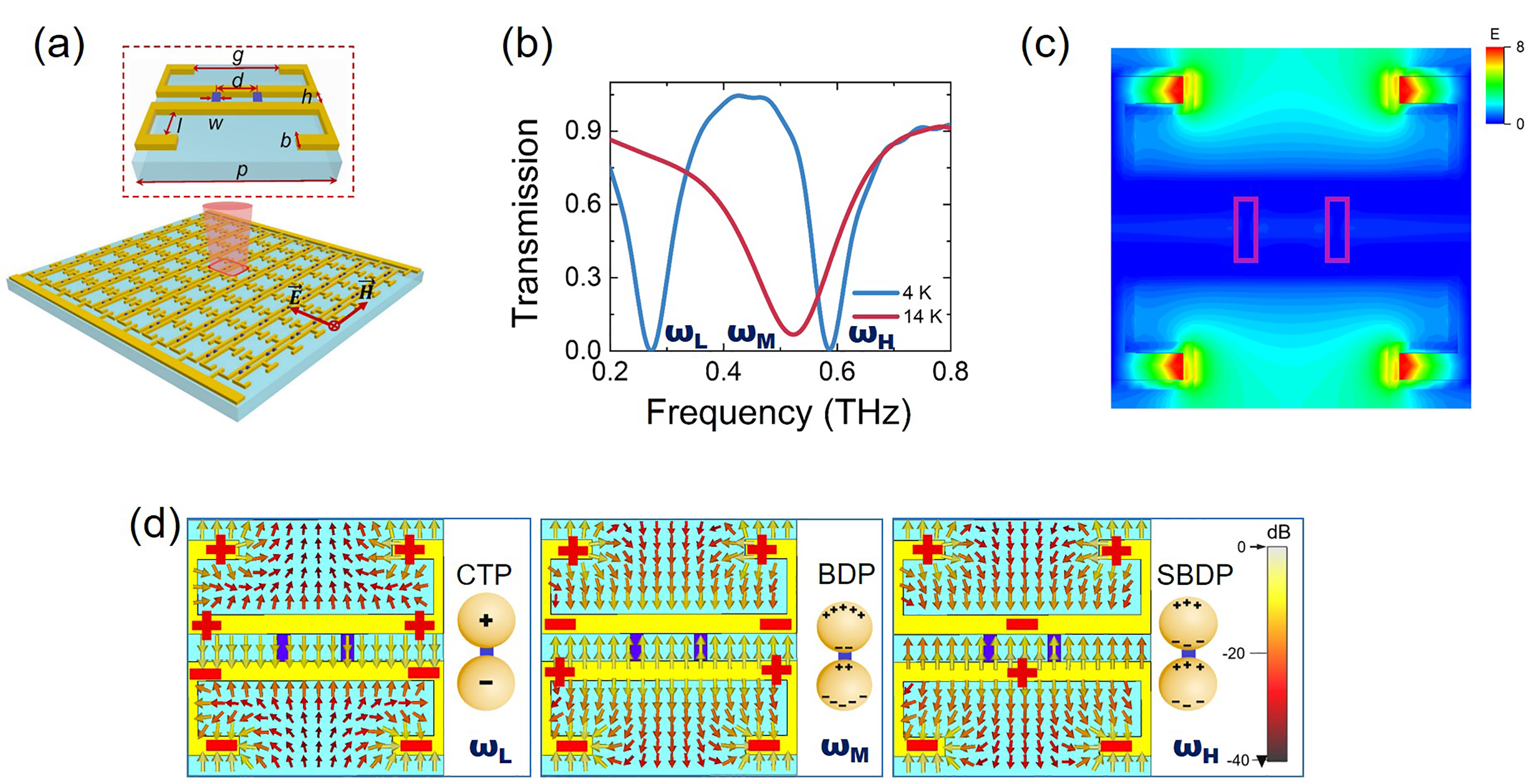


**Figure S1.** Diagram and simulated spectra of the hybrid metasurface. (a) Schematic diagram of NbN-Au hybrid metasurface. The close-up shows the unit cell with geometrical parameters: *w* = 6 μm, *b* = 9 μm, *h* = 13 μm, *l* = 26 μm, *d* = 30 μm, *g* = 72 μm, and *p* = 120 μm. (b) Simulated THz transmission spectra under different temperatures. (c) Simulated normalized electric field distribution in a unit cell of the hybrid metasurface. The two purple rectangular boxes correspond to the NbN microbridges. (d) Distributions of the electric field at the resonance frequencies of 0.28 THz (*ω*_L_), 0.52 THz (*ω*_M_), and 0.59 THz (*ω*_H_), corresponding to CTP, BDP, and SBDP mode, respectively.

The schematic of the NbN-Au hybrid metasurface is shown in Figure S1(a). The close-up shows the unit cell with geometrical parameters: *w* = 6 μm, *b* = 9 μm, *h* = 13 μm, *l* = 26 μm, *d* = 30 μm, *g* = 72 μm, and *p* = 120 μm. The simulated electric field distribution is shown in Figure S1(d). In the superconducting state, the microbridges act as conductive channels, with charges flowing through them. At *ω*_L_, the positive and negative charges are accumulated in the upper and lower rings, respectively, inducing an electric dipole resonance. The mode is similar to the charge transfer plasmonic (CTP) mode in the plasmonic dimer [S1]. For *ω*_H_, each part of the upper and lower rings has positive and negative charges. Consequently, the electric field in the center of the gap is shielded. This mode is named the screened BDP (SBDP) mode. In the normal state, the NbN microbridge is lossy, and the conductive coupling is suppressed. As seen in the electric field distribution at the frequency of *ω*_M_, the charge density is highest at the upper and lower ends of the unit cell structure. The resonance mode is the hybrid mode of two electric dipole modes, similar to the bonding dimer plasmonic (BDP) in the plasmonic dimer. Figure S1(c) shows the simulated electric field distribution using electromagnetic simulation software. The simulated electric field is normalized by the electric field of the bare substrate at 0.55 THz. The two rectangular boxes with purple borders correspond to NbN microbridges. The normalized electric field around the two regions is near 1, indicating no significant field enhancement around the NbN microbridges of the hybrid structure.

**Supplementary Note 2 Pump-probe THz spectroscopy system**

A cryogenic pump-probe THz spectroscopy system is used for the THz pump-THz probe and optical pump-THz probe measurement. The optical path diagram is shown in Figure S2. The femtosecond laser pulse is generated from a titanium sapphire femtosecond pulse regenerative amplifier laser with a repetition frequency of 1 kHz, a maximum output power of 6 W, and a center wavelength of 800 nm. The THz probe pulses are generated using a 1 mm thick ZnTe crystal. The tilted-pulse-front optical rectification in LiNbO_3_ crystal is utilized to generate intense THz pump pulses [S2]. Free-space electro-optical sampling is used in the ZnTe crystal to detect the temporal waveform of the transmitted probe pulse. A wire grid polarizer (WG_1_) is used to change the field strength of the THz pump pulse. WG_2_ is used to reflect THz probe pulses and transmitted pump pulses. WG_3_ is placed behind the sample to block the THz pump pulse. The maximum electric field of THz pulses is 25 kV/ cm. In our setup, the measurement configuration can be switched between the optical pump-THz probe and THz pump-THz probe measurement by inserting a switching mirror.


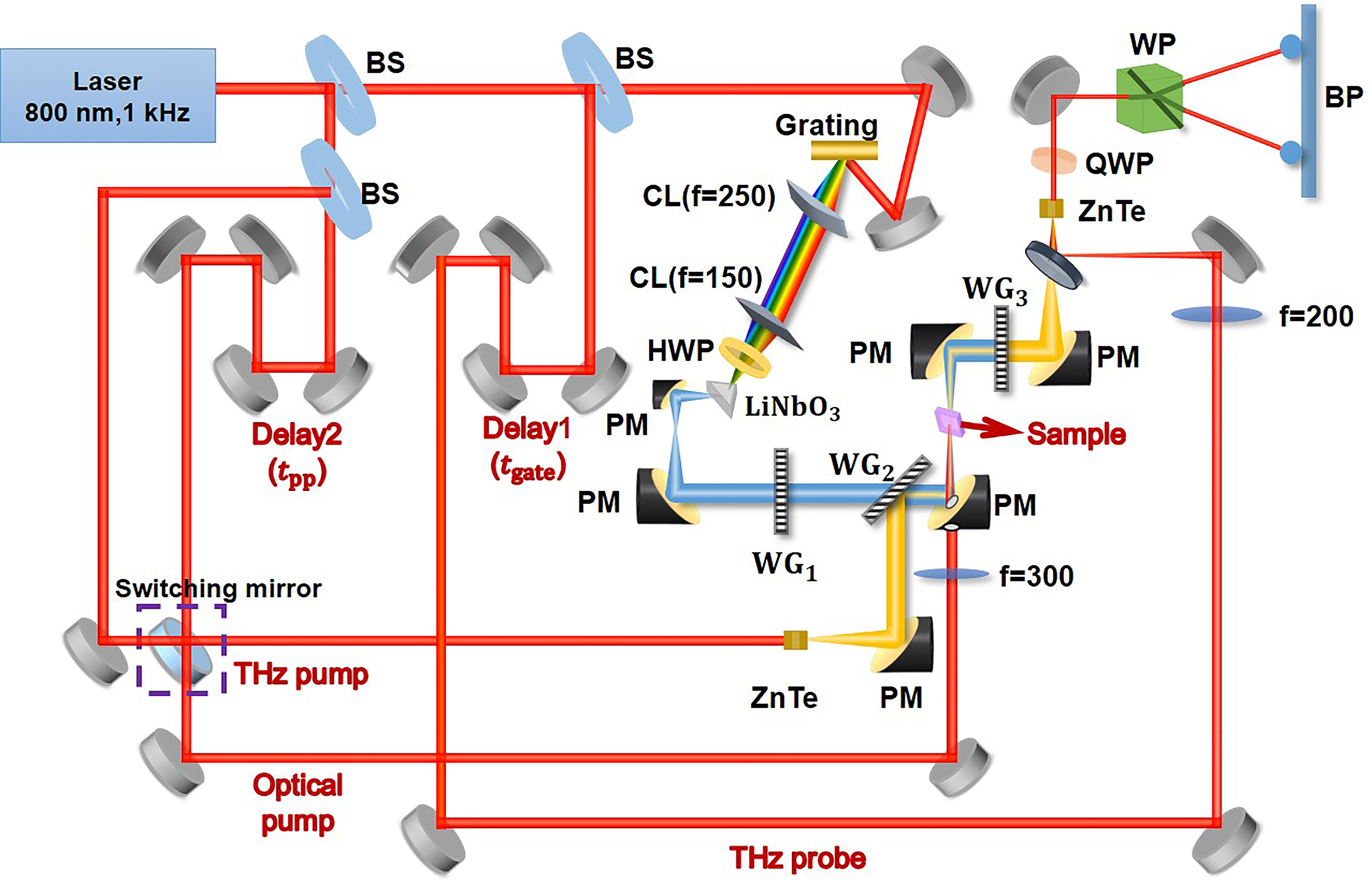


**Figure S2.** Diagram of pump-probe THz spectroscopy system. BS: beam splitter, CL: cylindrical lens, HWP: half-wave plate, PM: parabolic mirror, WG: wire grid polarizer, QWP: quarter-wave plate, WP: Wollaston prism, BP: balanced photodiode.

**Supplementary Note 3 Relationship between conductive coupling and mode switching in hybrid metasurface**

For the NbN-Au hybrid metasurface, the conductive coupling between two resonators is critical for mode switching [S3]. We analyzed the conductance threshold of mode switching by numerical simulation. In the device design, we varied the thickness of microbridges for simulation. Thus, the microbridge conductance could be changed. The simulated transmission spectra for different microbridge conductance in the normal state are shown in Figure S3(a). As the thickness decreases, the transition from dual resonance modes to single resonance modes appears in the simulated THz transmission spectra. The resonance frequencies in the transmission spectra as a function of microbridge conductance are shown in Figure S3(b). The charge flow between the upper and lower resonators results in the formation of the CTP resonance mode. The coupling between two resonators gradually weakens with the decrease of the conductance of the microbridge. When the conductance is lower than 0.12 S, the significant Ohmic loss of the NbN conductive channel leads to the disappearance of the CTP mode.


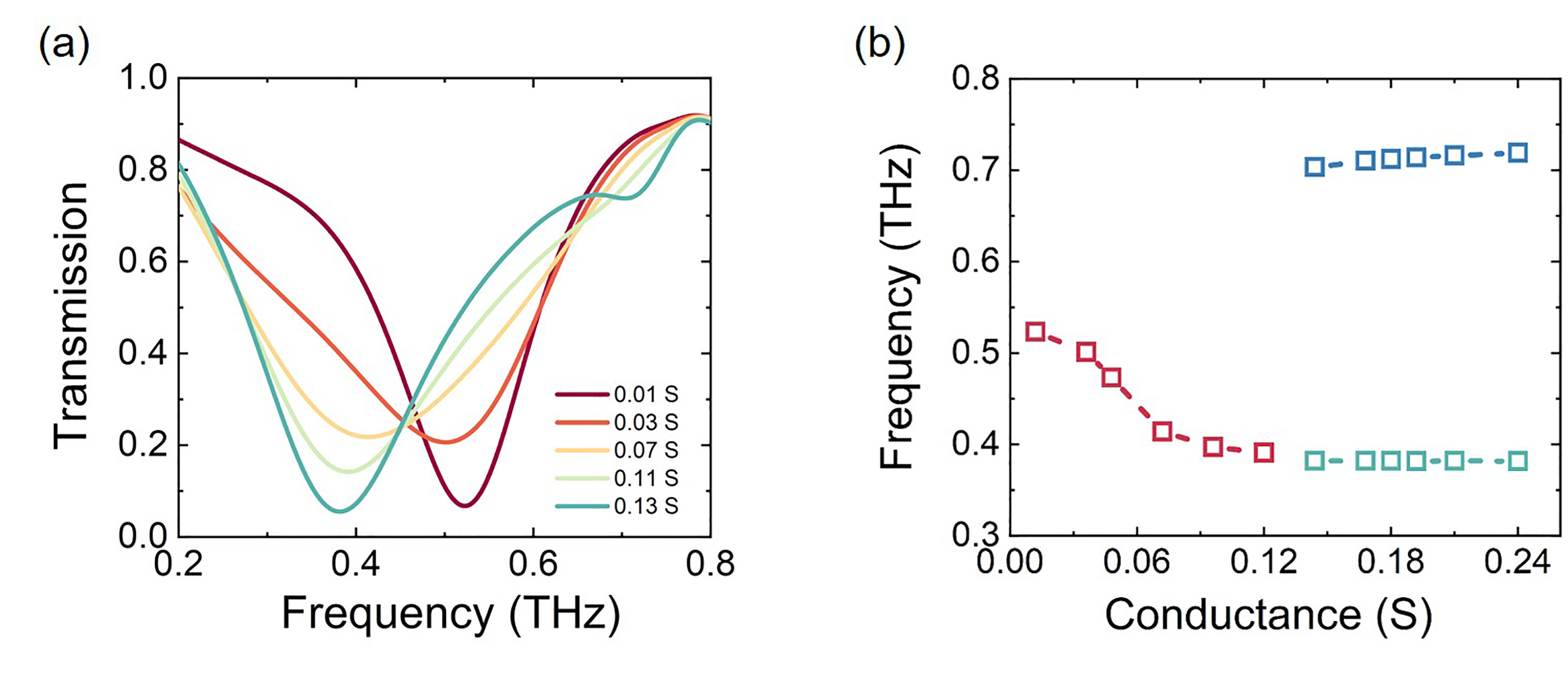


**Figure S3.** Dependence of mode switching in hybrid metasurface on conductive coupling. (a) Simulated THz transmission spectra for different thicknesses of NbN microbridge. (b) Resonance frequencies as a function of the microbridge conductance.

**Supplementary Note 4 Frequency tuning and transmission modulation of the THz hybrid metasurface**

The measured THz transmission spectra of the hybrid metasurface at different temperatures are shown in Figure 1(c) of the main article. As the temperature increases, the two resonance frequencies gradually redshift. The resonance frequency of CTP mode changes from 0.32 THz at 4 K and 0.25 THz at 13 K. Meanwhile, the resonance frequency of SBDP mode changes from 0.62 THz at 4 K to 0.53 THz at 13 K, as shown in Figs. S4(a) and S4(b).


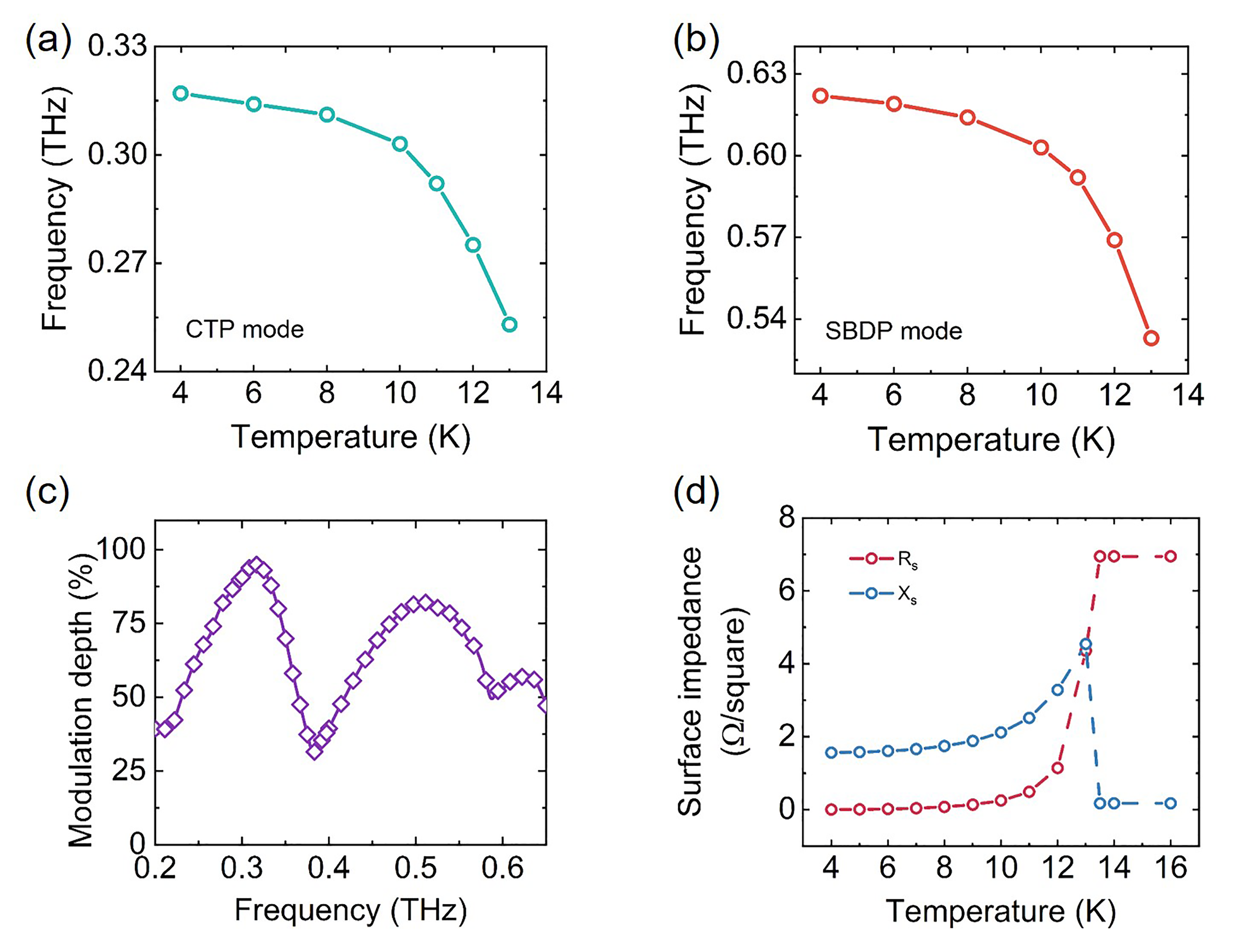


**Figure S4.** Temperature dependence of the measured THz transmission spectra. Measured resonant frequencies of CTP mode (a) and SBDP mode **(**b) as a function of temperature. (c) Calculated modulation depth spectra. (d) Complex surface impedance of 12 nm thick NbN film at 0.32 THz at different temperatures.

The calculated modulation depth from 0.2 THz to 0.65 THz is shown in Figure S4(c). The modulation depth (*η*) is defined as *η* = (|*T*_max_ – *T*_min_|)/ *T*_max_, where *T*_max_ and *T*_min_ are the maximum and minimum transmission coefficients at each frequency, respectively. The frequency tuning property significantly broadens the operating bandwidth of the hybrid metasurface as an electric modulator. A modulation depth of higher than 40% was obtained from 0.2 to 0.65 THz.

To explain the mode switching and frequency tuning behaviors of the hybrid metasurface, we calculated the complex surface impedance of 12 nm thick NbN film using the following formula [S4, S5]

$Z_{s}=R_{s}+jX_{s}=\sqrt{j\omega\mu_{0}/\sigma(\omega)}\coth\left( d\sqrt{j\omega\mu_{0}\sigma\left( \omega\right)} \right)$, (S1)

where *d* is the thickness of film and *μ*_0_ is the vacuum permeability. The complex surface impedance of the film at 0.32 THz at different temperatures is shown in Figure S4(d). When the temperature is 4 K, the *R_s_* of the film at 0.32 THz is close to 0.002 Ω/s, which is significantly lower than the value of *X_s_*. In the superconducting state, the reactive component dominates and varies with temperature. On the contrary, the microbridge can be equivalent to a resistor when the temperature is above 13.5 K. Thus, the complex impedance of superconducting film experiences drastic changes, leading to a significant difference in the conductance coupling between the two metallic resonators. When the microbridges become lossy in the normal state, the charge flow between two metallic resonators is nearly cut off, resulting in mode switching. The resonance frequency *ω*_0_ is determined by

$\omega_{0}={(\sqrt{(L_{g}+L_{k})C})}^{-1}$, (S2)

where *C* is the effective capacitance, *L_g_* is the geometry inductance, and *L_k_* is the kinetic inductance. For superconducting films, the relationship between *L_k_* and *X_s_* satisfies *X_s_* = ω*L_k_*. Therefore, the *L_k_* of the NbN film in the superconducting state increases gradually with temperature, which causes a redshift in the resonance frequency.

**Supplementary Note 5 Ultrafast mode switching triggered by optical pulse pump**

The mapping of the measured electric field of the THz probe pulse (*E*_probe_) when the optical pump fluence is 6.4 μJ/cm^2^ is displayed in Figure S5(a). When taking the electric field of *t*_pp_ = –3.0 ps as the reference, we calculated the change of *E*_probe_ (Δ*E*_probe_) as a function of *t*_pp_. As shown in Figure S5(b), there are remarkable changes in *E*_probe_ when *t*_pp_ > 0 ps. The measured THz probe pulses at *t*_pp_ of –2.0, 2.0, and 6.0 ps as shown in Figure S5(c), correspond to the dashed lines marked in Figure S5(a). The electric field changes significantly at *t*_gate_ = 6.7 ps, while the amplitude of the main pulse remains roughly the same. The Δ*E*_probe_ for the transmitted THz pulse as a function of *t*_pp_ when *t*_gate_ = 6.7 ps under different optical pump fluences is plotted in Figure S2(d). We defined the time that the transmitted THz pulse starts changing as *t*_pp_ = 0 ps. Δ*E*_probe_ increases after the arrival of the optical pump, and the rising time is several picoseconds. Furthermore, the rising time shows dependence on the pump fluence. With the increase of the pump fluence, the amplitude of Δ*E*_probe_ increases, and the rising time become shorter [S6].


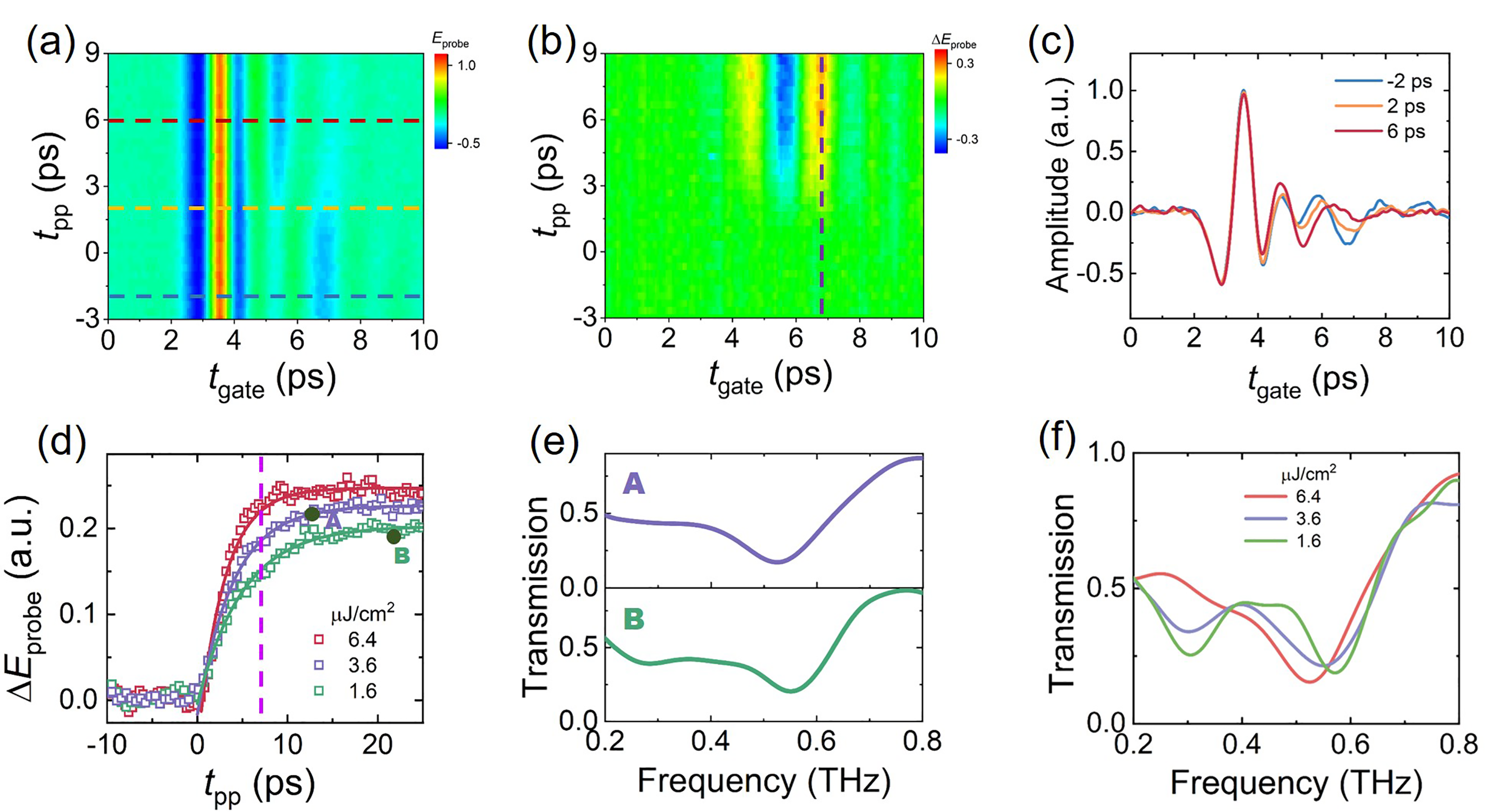


**Figure S5.** Measured time-domain profiles and frequency spectra of the transmitted THz pulse under optical pump stimulation. Mapping of the measured electric field of the THz probe pulse (*E*_probe_) (a) and change in *E*_probe_ (Δ*E*_probe_) (b) as a function of pump-probe delay (*t*_pp_) when the optical pump fluence is 6.4 μJ/cm^2^. (c) Measured time-domain profiles of the THz probe pulses at *t*_pp_ = –2.0, 2.0, and 6.0 ps. The data are corresponding to the dashed lines marked in (a). (d) Measured *ΔE*_probe_ (squares) using the optical pump-THz probe system as a function of *t*_pp_ under various pump fluence (*t*_gate_ = 6.7 ps). The data correspond to the dashed lines marked in (b). Solid lines represent the fitting curves. A and B denote the positions of *t*_pp_ that the transition process is finished. The corresponding measured transmission spectra are plotted in (e). (f) Measured THz transmission spectra under different pump fluences when *t*_pp_ = 7.0 ps. The data are corresponding to the dashed lines marked in (d).

To quantitatively analyze the difference under different pump fluences, we use the following formula to fit the curve of Δ*E*_probe_,

$s= s_{0}+A*exp(-(t-t_{0})/\tau)$, (S3)

where *s*_0,_ *t*_0,_ and *A* are constant, and *τ* is the decay constant. As shown in Figure 2(e), *τ* monotonously increases with the pump fluence. The values of *τ* are 2.9, 3.9, and 5.0 ps for the pump fluences of 6.4, 3.6, and 1.6 μJ/cm^2^, respectively. The calculation of the dynamic process based on the Rothwarf–Taylor model efficiently explained the transition behavior of Δ*E*_probe_ and the pump intensity dependence of the hybrid metasurface [S7].

Figure S5(e) shows the transmission spectra under different pump fluence when *t*_pp_ is 13 and 21 ps, corresponding to A and B in Figure S5(d). When the pump fluence is 3.6 μJ/cm^2^, the mode switching has been completed at *t*_pp_ = 13.0 ps, and there is only one resonant mode in the measured transmission spectra. When the pump fluence is 1.6 μJ/cm^2^, we can see two resonant modes when the transition process is finished (*t*_pp_ = 21 ps). When *t*_pp_ is 7.0 ps, the transmission spectra under different pump fluence as shown in Figure S5(f). The data correspond to the dashed lines in Figure S5(d). These results indicate that the switching time and the spectral responses are tunable by the optical pump fluence [S8].

**Supplementary Note 6 Time-domain pulse of THz pump and corresponding Fourier transform spectra under different pump filed strength**

For the THz pump-THz probe experiment, the time-domain profiles and frequency spectra of the THz pump pulses used with different peak electric field strengths are plotted in Figure S6. The width of the THz pump pulse is about 3 ps, and the effective bandwidth is 0.2–1.2 THz. The maximum pump THz electric field strength (*E*_0_) is 25 kV/cm.


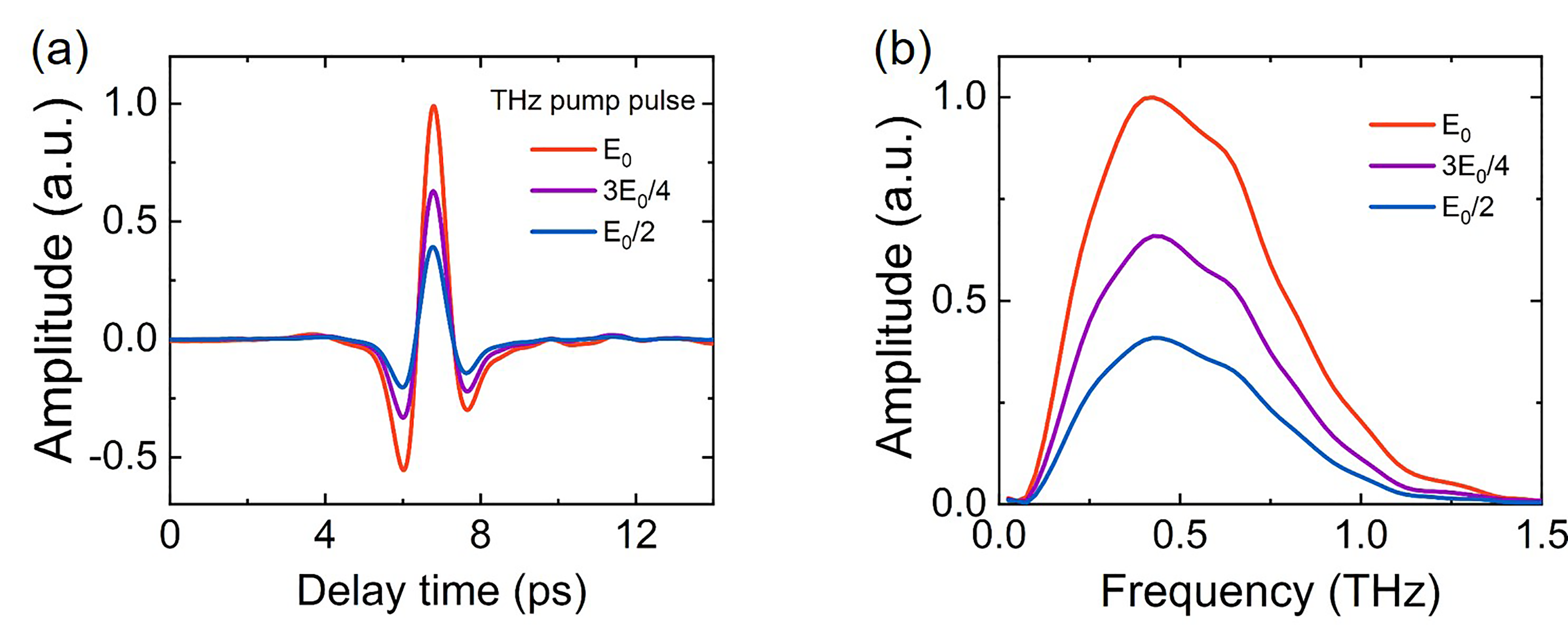


**Figure S6.** (a) Time-domain profiles and (b) frequency spectra of the THz pump pulses with different peak electric field strengths.

**Supplementary Note 7 Time evolution of Δ*E*_probe_ under different THz pump strength**

Taking the electric field of *t*_pp_ = –2.0 ps as the reference, we calculated Δ*E*_probe_ as a function of *t*_pp_ when the THz pump field strength is *E*_0_, as shown in Figure S7(a). There are two apparent changes in the transmitted THz electric field appearing at 5.7 and 7.0 ps, respectively. The temporal evolution of Δ*E*_probe_ (*t*_gate_ = 5.7 ps) at 4 K for various THz pump strengths is displayed in Figure S7(b). There are apparent oscillations in the time windows between *t*_pp_ = 3.0 and 6.0 ps. The oscillation strength shows the dependence on the peak electric field strength. These phenomena are in accordance with the measured temporal evolution of Δ*E*_probe_ when *t*_gate_ = 7.0 ps, as shown in Figure 4(a) of the main article. The oscillation of Δ*E*_probe_ with *t*_pp_ remains the same for different *t*_gate_, and it further proves that the oscillations originate from the Higgs mode oscillation [S9-S11].


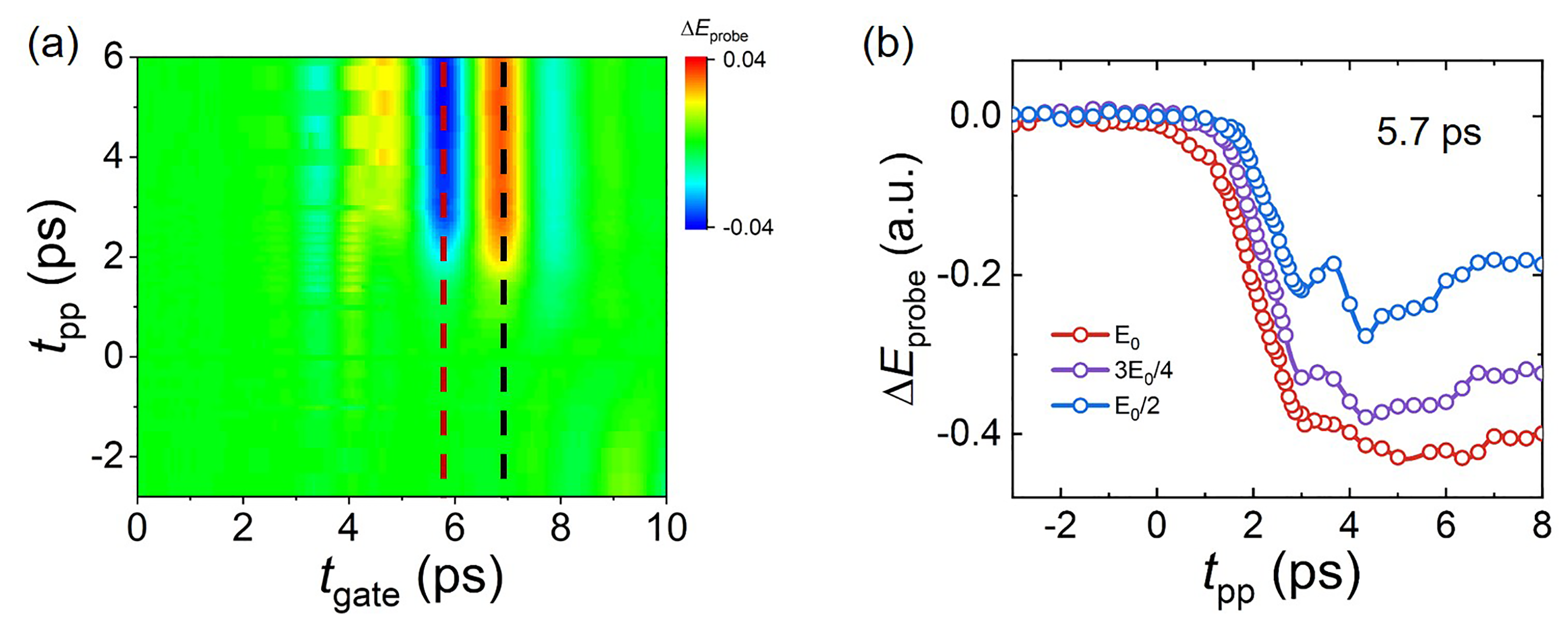


**Figure S7.** Time evolution of Δ*E*_probe_ under the THz pump. (a) Mapping of Δ*E*_probe_ as a function of *t*_pp_ when the pump THz field strength is *E*_0_. (b) Temporal evolution of Δ*E*_probe_ (*t*_gate_ = 5.7 ps) at 4 K for various THz pump intensities. Data correspond to the red dashed lines marked in (a).

**Supplementary Note 8 Dynamic transition process of THz transmission spectra and the change of transmission amplitude with *t*_pp_ under different THz pump strengths**

At pump field strength of 3*E*_0_/4, the dynamic transition process of THz transmission spectra when *t*_pp_ is between 0 and 6.0 ps is shown in Figure S8(a). The change in the transmission (Δ*T*) at 0.32, 0.61, and 0.5 THz shows oscillations in the time window between *t*_pp_ = 3.0 to 6.0 ps, as shown in Figure S8(b). Similar to the experimental results with the minimum field strength of 0.5*E*_0_ demonstrated in the main article, the transmission amplitude at the resonance frequencies likewise exhibits oscillation behavior.

When the pump strength is *E*_0_, the dynamic transition process of THz transmission spectra is shown in Figure S8(c), Δ*T* also oscillates at 0.3 and 0.6 THz, as shown in Figure S8(d). It can be seen that the oscillation of Higgs amplitude mode shows the pump field strength dependence, and it is the least apparent when the pump field strength is *E*_0_. The increase of the THz pump field strength results in more Cooper pairs breaking. The superconductivity is significantly suppressed under the pump field strength of *E*_0._ Correspondingly, the oscillations of the superconducting order parameter are not significant. As shown in Fig. 4 and Fig. S8, the stronger Higgs mode oscillations are found to lead to more pronounced modulation effects of the transmission amplitude around the resonance frequencies.


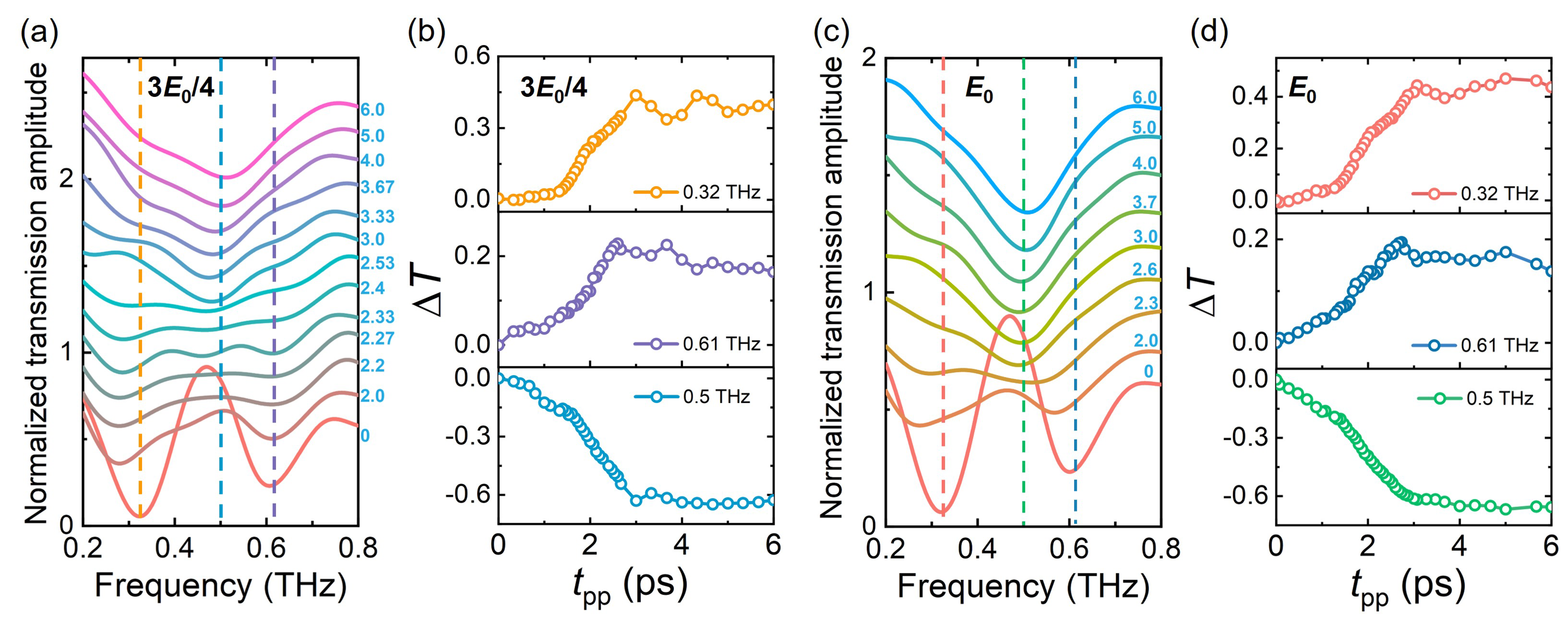


**Figure S8.** Measured transmission spectra and transmission change as a function of *t*_pp_ under pump intensity of 3*E*_0_/4. Dynamic transition process of THz transmission spectra under pump intensity of 3*E*_0_/4 (a) and *E*_0_ (c) (each curve is offset vertically by 0.15). Measured Δ*T* (take 0 ps as a reference) at 0.32, 0.61, and 0.5 THz as a function of *t*_pp_ when the pump field strength is 3*E*_0_/4 (b) and *E*_0_ (d).

**Supplementary Reference**

[S1] O. Pérez-González, N. Zabala, A. G. Borisov, et al., "Optical Spectroscopy of Conductive Junctions in Plasmonic Cavities," *Nano Lett.*, vol. 10, pp. 3090-3095, 2010.

[S2] H. Hirori, A. Doi, F. Blanchard, K. Tanaka, "Single-cycle terahertz pulses with amplitudes exceeding 1 MV/cm generated by optical rectification in LiNbO_3_", *Appl. Phys. Lett.*, vol. 98, pp. 091106, 2011.

[S3] H. Zhang, C. Li, C. Zhang, et al., "Experimental study on the transition of plasmonic resonance modes in double-ring dimers by conductive junctions in the terahertz regime," *Opt. Express*, vol. 24, pp. 27415-27422, 2016.

[S4] H.-T. Chen, H. Yang, R. Singh, et al., "Tuning the Resonance in High-Temperature Superconducting Terahertz Metamaterials," *Phys. Rev. Lett.*, vol. 105, pp. 247402, 2010.

[S5] J. Wu, B. Jin, Y. Xue, et al., "Tuning of superconducting niobium nitride terahertz metamaterials," *Opt. Express*, vol. 19, pp. 12021-12026, 2011.

[S6] R. Matsunaga, and R. Shimano, "Nonequilibrium BCS State Dynamics Induced by Intense Terahertz Pulses in a Superconducting NbN Film," *Phys. Rev. Lett.*, vol. 109, 187002, 2012.

[S7] M. Beck, M. Klammer, S. Lang, et al., "Energy-Gap Dynamics of Superconducting NbN Thin Films Studied by Time-Resolved Terahertz Spectroscopy," *Phys. Rev. Lett.*, vol. 107, pp. 177007, 2011.

[S8] Y. K. Srivastava, M. Manjappa, L. Cong, et al., "A Superconducting Dual-Channel Photonic Switch," *Adv. Mater.*, pp. 1801257, 2018.

[39] Y. K. Srivastava, M. Manjappa, H. N. S. Krishnamoorthy, et al., "Accessing the High-Q dark Plasmonic Fano Resonances in Superconductor Metasurfaces," *Adv.Opt. Mater*, vol. 4, pp. 1875-1881, 2016.

[S9] R. Matsunaga, and R. Shimano, "Nonlinear terahertz spectroscopy of Higgs mode in s-wave superconductors," *Phys. Scr.*, vol. 92, pp. 024003, 2017.

[S10] R. Matsunaga, and R. Shimano, "Higgs amplitude mode in s-wave superconductors revealed by terahertz pump-terahertz probe spectroscopy," presented at the *Proc. SPIE*, vol. 9361, pp. 93611D, 2015.

[S11] M. K. Rafailov, E. Mazur, R. Matsunaga, et al., "Higgs mode excitation in superconductors by intense terahertz pulse," presented at the *Proc. SPIE*, vol. 9835, pp. 98351G, 2016.
